# Bioactive Flavanone Glycoside Isolated from Leaves of *Faramea* Species Presents Antiviral and Protective Activity against Zika and Mayaro Virus Infection

**DOI:** 10.1021/acsomega.5c06047

**Published:** 2025-12-03

**Authors:** Iris Paula Guimarães-Andrade, Rodolfo Santos Barboza, Mariana Oliveira Lopes da Silva, Raissa Alves da Conceição, Nathalia Carraio de Albuquerque, Daniel Gavino-Leopoldino, Rômulo Leão Silva Neris, Alessandra Mendonça Teles de Souza, Ligia Maria Marino Valente, Iranaia Assunção-Miranda

**Affiliations:** † LaRIV, Department of Virology, Instituto de Microbiologia Paulo de Góes, 28125 Universidade Federal do Rio de Janeiro (UFRJ), Rio de Janeiro 21941-902, Brazil; ‡ Instituto de Química, Universidade Federal do Rio de Janeiro (UFRJ), Rio de Janeiro 21941-909, Brazil; § Laboratory of Molecular Modeling & QSAR Faculty of Pharmacy, Universidade Federal Do Rio de Janeiro, Rio de Janeiro, Rio de Janeiro 21944-970, Brazil

## Abstract

The high frequency of outbreaks, territorial expansion, and cocirculation of different arboviruses reinforce the necessity of efforts for the development of prevention and therapeutic strategies. We previously characterized an anti-Dengue virus activity of 2S-5-hydroxy-4′-methoxy-flavanone-7-*O*-β-D-apiofuranosyl-(1→6)-β-d-glucopyranoside, a bioactive flavanone glycoside (FvGly) that could be isolated from leaves of different *Faramea* species. Here, we investigated the ability of FvGly to inhibit other arboviruses, testing against Zika virus (ZIKV), Mayaro virus (MAYV), and Chikungunya virus infections. We demonstrated that FvGly inhibits ZIKV and MAYV infectious particle release from neuronal (SH-SY5Y) and skeletal muscle (C2C12) cells, respectively, when treated before and after viral infection, without presenting a virucidal effect. Treatment with FvGly also results in protection from virus-induced cell death. In agreement, treatment with FvGly reduced the ZIKV and MAYV RNA loads released after infection, and the expression of envelope protein was tightly inhibited in MAYV-infected C2C12 cells. To get insights about the FvGly mechanism, we used target fishing comparative analysis that predicted iNOS, Akt1, and Mapk14 as targets. Molecular docking also supported the high-affinity binding of FvGly with selected targets, mainly iNOS. Finally, we tested the effect of daily treatment with FvGly in a mouse model of MAYV-induced disease. We observed that treatment reduced clinical signs and muscle lesions compared to untreated infected mouse groups. Taken together, our data indicate that, in addition to Dengue, FvGly treatment also has an antiviral and protective effect against ZIKV and MAYV infection.

## Graphical abstract

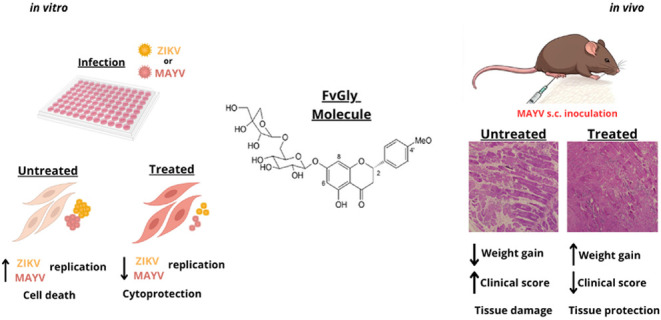

## Introduction

Diseases caused by arboviruses such as Dengue (DENV), Chikungunya (CHIKV), Zika (ZIKV), Yellow Fever (YFV), and West Nile virus (WNV) represent a significant global health problem.^1–4^ The spread of these arboviruses is notorious, as recent cases of Mayaro virus (MAYV) and Oropouche virus (OROV), both previously restricted to forest regions of the Amazon basin, are now circulating in urban areas, with increasing outbreaks reported.^5^ High frequency of outbreaks, together with the risk of emergence and reemergence of arboviruses with pandemic potential, reinforces the urgency of raising efforts for improving viral surveillance, vector control, and developing efficient prevention and therapeutic strategies.^6^

Currently, there are no available antiviral treatments targeting those viruses. Despite progress, vaccination against arboviruses still faces challenges in containing new outbreaks, such as the YFV outbreak in Brazil in 2016.^7^ This highlights the importance of investigations of new antiviral compounds, especially those with broad-spectrum activity against arboviruses. The research for compounds capable of acting on multiple targets, in addition to being economically favorable, is an interesting strategy due to the high cocirculation and overlapping of symptoms from the different arboviruses.^8^ Natural resources have been shown to be an excellent reservoir for active molecules that could be used to target many different diseases and pathogens.^9,10^ Flavonoids are a huge family of polyphenolic multibioactive molecules produced by plant metabolism, whose properties have been extensively studied, including anti-inflammatory, antioxidant, and antiviral activities against a wide range of viral infections, representing promising molecules.^11–16^

The genus *Faramea* Aubl. (Rubiaceae) is native but nonendemic to Brazil and contains species in the form of shrubs, subshrubs, or trees. This is a neotropical genus with about 200 species, of which 123 are found in Brazil, distributed along humid tropical areas of the coast and the Atlantic Forest.^17^ Despite the high diversity of species that compose the *Faramea* genus, limited data can be found about its chemical composition and pharmacological potential. Previous studies by our group isolated and characterized a new bioactive flavanone glycoside (FvGly), 2S-5-hydroxy-4′-methoxy-flavanone-7-*O*-β-D-apiofuranosyl-(1→6)-β-d-glucopyranoside, with anti-DENV2 activity.^18^ The FvGly was first isolated from the leaves of *F. bahiensis*, but could also be found in *F. truncata* and *F. hyacinthina*, all endemic to Brazil.^18,19^

Here, we investigated the ability of FvGly, in addition to controlling DENV infection, to have broad-spectrum activity against other arboviruses of medical importance. Thus, using *in vitro* cultures of target cells, and also *in silico* and *in vivo* approaches, we demonstrated that FvGly treatment has antiviral and protective activity in MAYV and ZIKV infections. Our data reinforce the potential use of this class of natural molecules as a template for the development of new and more efficient compounds targeting arboviruses.

## Results

### FvGly Inhibits ZIKV and MAYV Replication

In the present study, we used FvGly isolated from leaves of *F. truncata* (Figure 1A) and determined its cytotoxicity on cell cultures of SH-SY5Y (Figure 1B), a human neuroblastoma cell lineage used to study ZIKV replication,^20^ and C2C12 (Figure 1C), a murine myoblast cell lineage frequently used as a model of arthritogenic alphavirus replication.^21^ Neuronal and muscular cells were treated with vehicle or increasing concentrations of FvGly, and viability was determined by MTT assay 30 h post-treatment. FvGly showed low cytotoxicity for both cell cultures, even at the highest doses. Despite some effect in C2C12, no tested dose reduced cell viability by more than 20% compared to the non-treated control (Figure 1C). Thus, FvGly CC50 in both cell types was considered higher than 200 μg/mL (CC50 > 200 μg/mL).

**Figure 1:**
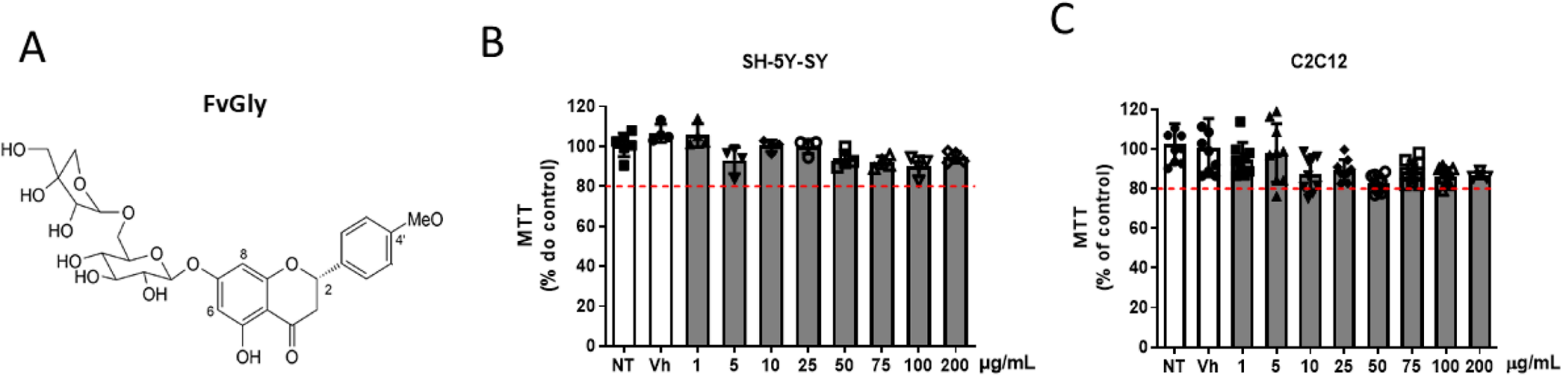
Structure and cytotoxicity of FvGly. (A) Molecular structure of FvGly isolated from *Faramea truncata*. (B,C) Stock solutions were prepared in dimethyl sulfoxide (DMSO), and indicated cell cultures were treated with increasing concentrations of FvGly. Cytotoxicity was determined by MTT assay. Red dotted lines indicate the established cutoff of 80% metabolization relative to NT or vehicle. The highest final concentration of DMSO was used as the vehicle (Vh) condition in the assays. NT: Not treated. Results are presented as means ± SD of data from three independent experiments.

To assess FvGly antiviral activity, we quantify infectious particle release after pre-infection treatment (Pre-T) or post-infection treatment (Pos-T) strategies. For ZIKV infection in SH-SY5Y, we observed that both pre- and post-infection treatment with 10 and 100 μg/mL of FvGly significantly reduced ZIKV infectious particle release, inhibiting it by up to 80% compared to infection in untreated conditions (Figure 2A). Cytoprotection was not observed in the Pre-T strategy, but we found a tendency to prevent neuronal cell death in Pos-T (Figure 2B). These results confirm that, in addition to DENV, FvGly is effective against other flaviviruses.

**Figure 2:**
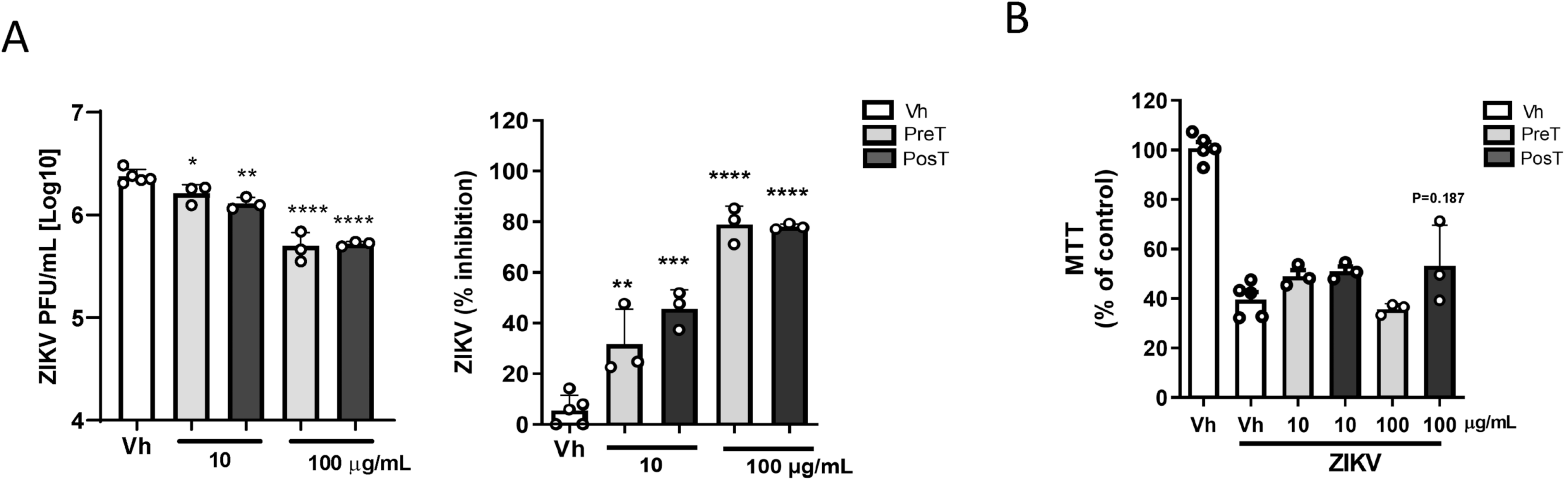
Treatment with FvGly inhibits ZIKV replication. Human neuroblastoma cell lineage (SH-SY5Y) culture was treated with FvGly before (Pre-T) or after (Pos-T) ZIKV infection. (A) Quantification of infectious particles released was determined by plaque assay, expressed in PFU/mL, and the effect of FvGly treatment was determined as % inhibition using the viral titer of the vehicle (Vh) condition as reference. (B) SH-SY5Y cell viability was determined by MTT assay after 48 h of ZIKV infection treated with Vh or FvGly in Pre-T and Pos-T strategies. Results are presented as means ± SD of data from three independent experiments. *p < 0.05, **p < 0.01, ***p < 0.001, ****p < 0.0001 relative to Vh. Statistical analysis was performed by one-way ANOVA followed by Tukey’s multiple comparison test.

Similar to ZIKV, MAYV release was significantly reduced when cells were pre- and post-infection treated with 100 μg/mL of FvGly (Figure 3A, B). In addition, using lower doses, we observed a dose-dependent inhibition, with the post-treatment strategy being more efficient, as it significantly reduced viral load even at 1 μg/mL of FvGly. To verify a possible additive effect, we combined both strategies (pre-infection and post-infection treatment), but the anti-MAYV activity of FvGly was not improved (Figure S1A). We also investigated the impact of FvGly treatment on the viability of infected cells. In agreement with the highest efficiency in controlling MAYV replication, FvGly significantly reduced cell death, mainly under the post-infection treatment condition (Figures 3C,D and S1B). Curiously, even though CHIKV belongs to the same genus as MAYV genus, post-treatment with FvGly did not efficiently reduce CHIKV release from infected C2C12 cells or viral-induced cell death (Figure S1C,E). A slight inhibition was only observed when we combined pre- and post-infection treatment, but it did not reduce cell death (Figure S1D,F). We also wondered whether this specific effect was related to a direct effect of FvGly on MAYV or ZIKV infectious particles, which might explain its selective activity. Nevertheless, direct incubation of CHIKV, MAYV, and ZIKV particles with FvGly did not alter viral infectivity (Figure S2). Together with the pretreatment effect, these data reinforce that FvGly may be interfering with some cellular pathway associated with viral replication in target cells.

**Figure 3:**
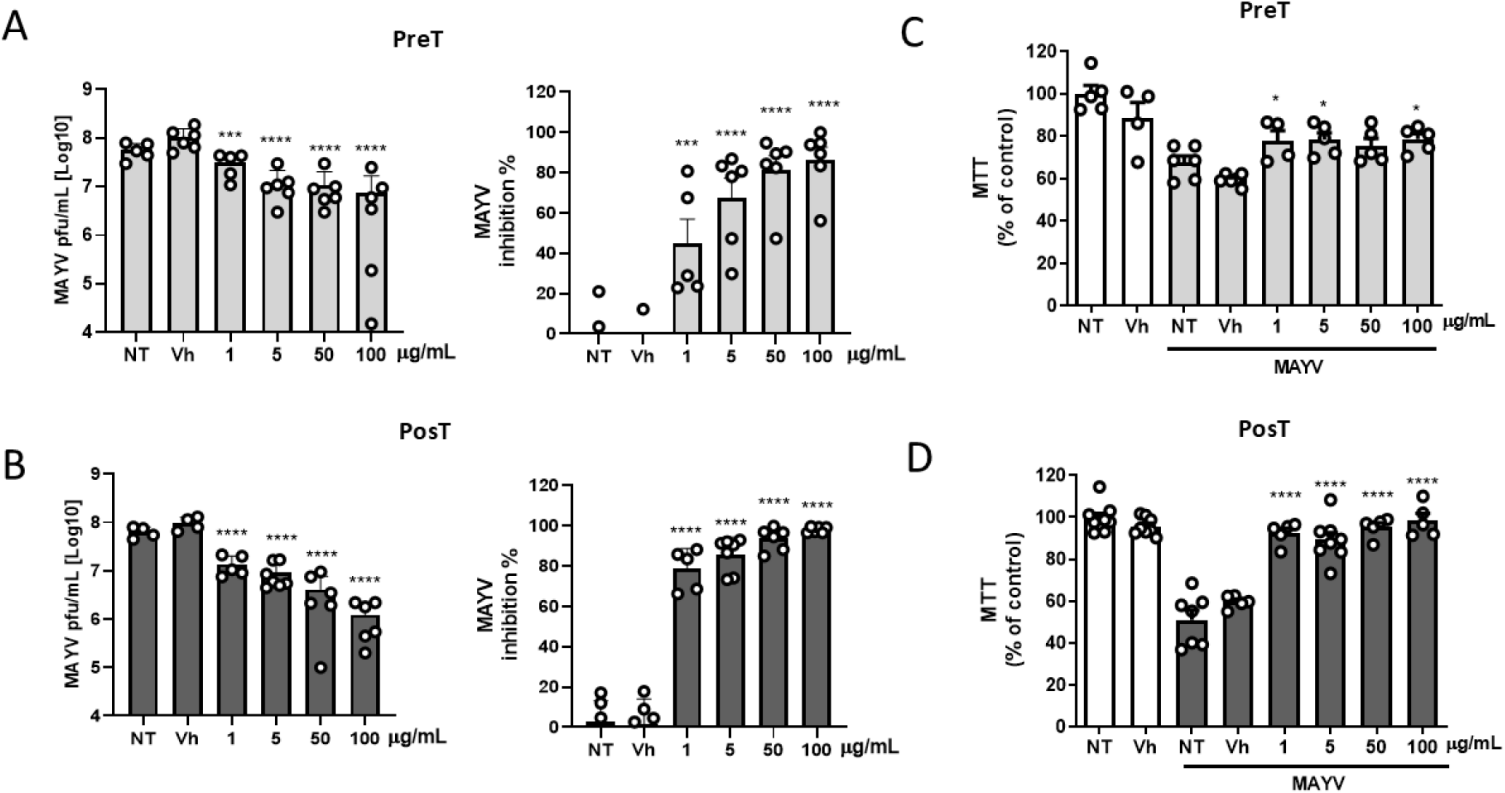
FvGly inhibits replication and reduces cell death induced by MAYV infection. Myoblast cell lineage (C2C12) culture was treated with increasing concentrations of FvGly before (Pre-T) or after (Pos-T) MAYV infection, as indicated at figure. (A, B) Quantification of infectious particles released was determined by plaque assay, expressed in PFU/mL, and the effect of FvGly treatment was determined as % inhibition using the viral titer of the vehicle (Vh) condition as reference. (C, D) C2C12 cell viability was determined by MTT assay after 48 h of MAYV infection treated with Vh or FvGly in Pre-T and Pos-T strategies. NT: Not treated. Results are presented as means ± SD of data from at least three independent experiments. Statistical analysis was performed using one-way ANOVA followed by Tukey’s multiple comparison test. *p < 0.05, ***p < 0.001, ****p < 0.0001 relative to Vh.

### The FvGly Treatment Reduces Viral RNA and Protein Expression in Target Cells

We followed by investigating whether FvGly could interfere with ZIKV and MAYV genomic RNA replication in cells. We tracked changes in intracellular and released RNA levels from infected cells under FvGly treatment (Figure 4). For ZIKV infection, the post-treatment with FvGly significantly reduced RNA released from SH-SY5Y cells after 72 h of infection, but surprisingly, it had only a slight impact on intracellular ZIKV RNA levels (Figure 4A). The same was observed in MAYV infection, where intracellular RNA levels were not altered in FvGly pre- nor post-treated C2C12-infected cells compared to untreated cells at 6 and 24 hpi (Figure 4B,D); however, we observed an important reduction in the RNA load released from infected cells using both treatment strategies (Figure 4C,E). Notably, lower levels of MAYV RNA in the supernatant were observed even at 6 hpi, a very early stage of infection in both strategies, indicating that although the treatment does not impact intracellular viral RNA, it could interfere with viral particle assembly or budding from infected cells.

**Figure 4:**
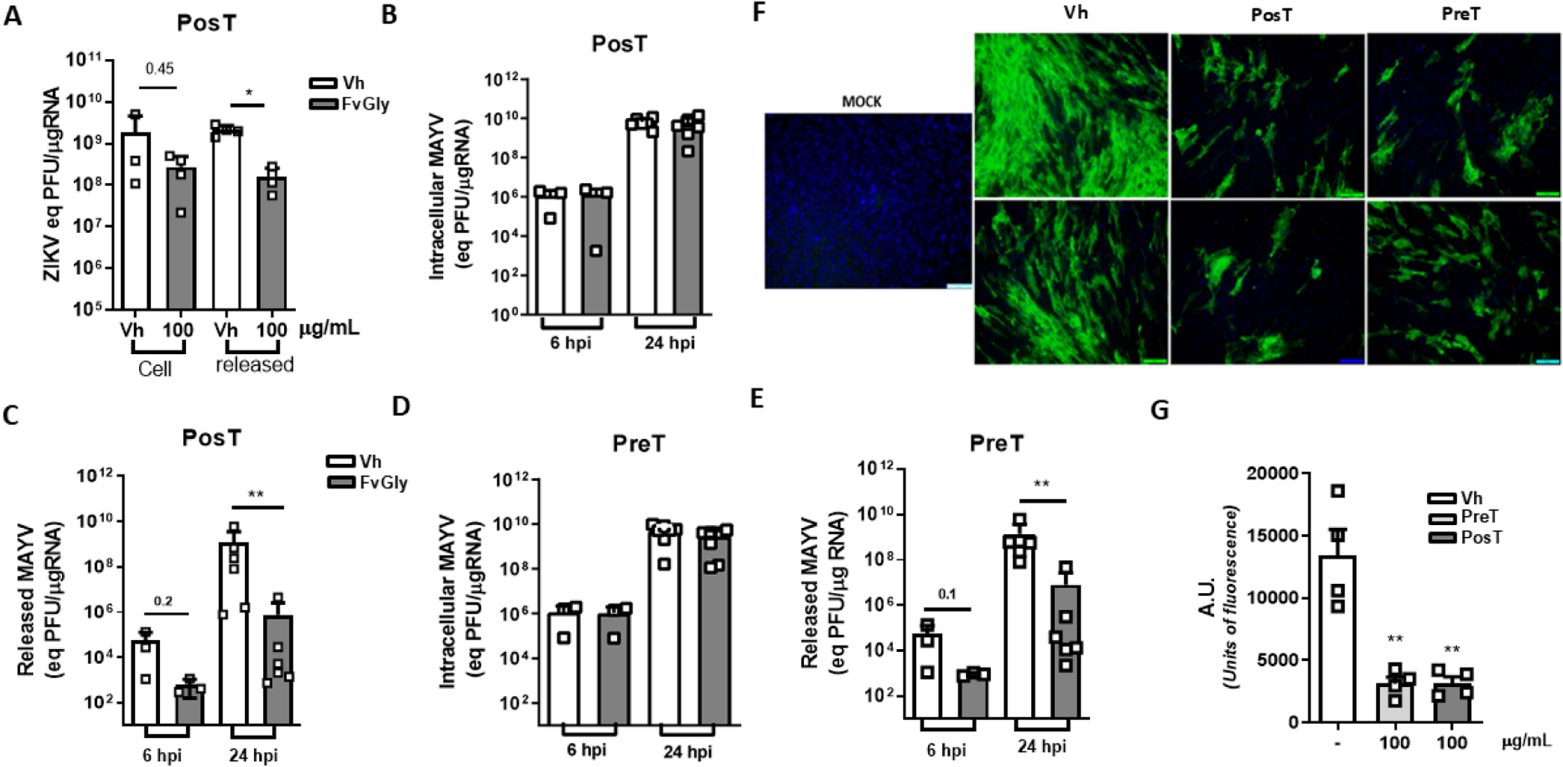
FvGly reduces viral RNA and protein expression in target cells. Human neuroblastoma cell lineage (SH-SY5Y) and myoblast cell lineage (C2C12) cultures were treated with 100 μg/mL of FvGly before (Pre-T) or after (Pos-T) ZIKV or MAYV infection. Genomic viral RNA loads were determined by RT-qPCR. (A) Intracellular (Cell) and released ZIKV RNA load from SH-SY5Y cells after 72 h of infection under FvGly or Vehicle (Vh) treatment in Pos-T strategy. (B) Intracellular or (C) released MAYV RNA load from C2C12 cells after 6 and 24 h post-infection (hpi) under FvGly or Vehicle (Vh) treatment in Pos-T strategy. (D) Intracellular or (E) released MAYV RNA load from C2C12 cells after 6 and 24 h post-infection (hpi) under FvGly or Vehicle (Vh) treatment in Pre-T strategy. (F) Immunofluorescence analysis for the detection of MAYV-positive C2C12 cells (green) at 30 hpi using antialphavirus E1 protein under FvGly or Vehicle (Vh) treatment in Pos-T and Pre-T strategies, as indicated in the figure. The cell nucleus was stained with DAPI (blue). (G) Quantitative analysis of fluorescence of E1-positive cells in images using ImageJ software. Results are presented as means ± SD of data from at least three independent experiments. Statistical analysis was performed using one-way ANOVA followed by Tukey’s multiple comparison test. *p < 0.05, **p < 0.01 relative to Vh.

To gather more evidence about the impact of treatment on viral replication, we performed indirect immunofluorescence staining of the MAYV envelope protein (E1) in untreated and treated C2C12-infected cells. We observed a marked reduction in E1 protein staining and the number of positive cells in the field when treated with FvGly using pre- and post-infection strategies in C2C12-infected cells (Figure 4F,G).

### *In Silico* Prediction of Cellular Target of FvGly

The antiviral FvGly effect with pre- and post-infection treatment strategies indicates that it may act on a cellular target important for viral replication. Thus, we used *in silico* target fishing, following molecular docking approaches, to predict the possible mechanism involved in FvGly’s anti-MAYV activity in cells. We used five servers: TargetHunter, SEA, BDB, and ChEMBL, which predict the targets based on ligand-based similarity searching, and PharmMapper, which identifies potential targets through receptor-based pharmacophore screening. Among these, three servers (TargetHunter, ChEMBL, and PharmMapper) retrieved potential targets for FvGly. To increase specificity, we overlapped the results, identifying 14 potential molecular targets that showed ligand structural similarity higher than 90% (Table S1). Three targets stand out by a direct protein–protein interaction network on the STRING: inducible nitric oxide synthase (iNOS), serine/threonine-protein kinase 1 (Akt1), and mitogen-activated protein kinase 14 (Mapk14) (Figure 5A). Molecular docking analyses were performed using 3D structures of iNOS, Akt, and MAPK to evaluate FvGly binding. The resulting complexes showed comparable binding profiles, mainly due to the hydrogen bond interactions between FvGly and iNOS (Figure 5B), Akt (Figure 5C), and Mapk14 (Figure 5D). The estimated binding energy values for the complexes (iNOS: −9.17 kcal/mol; Akt: −4.54 kcal/mol; Mapk14: −7.56 kcal/mol) supported the potential interaction of FvGly with these molecular targets, particularly with iNOS.

**Figure 5:**
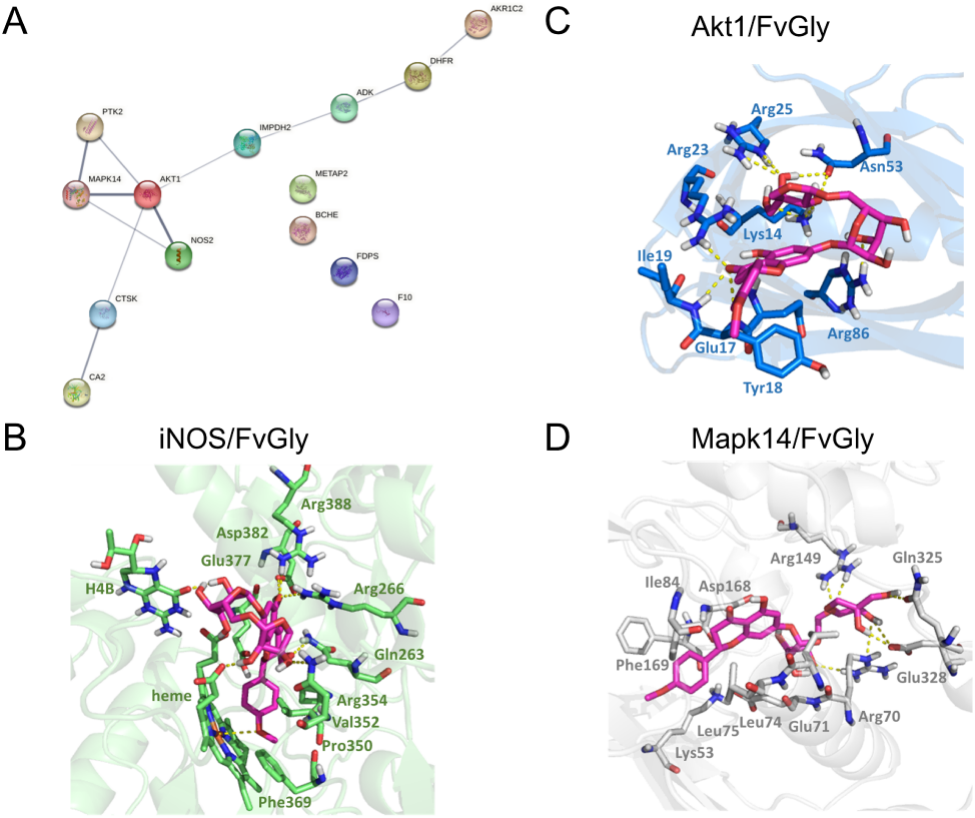
Prediction of the cellular target of FvGly using *in silico* analysis. *In silico* analysis was used to select and predict possible targets of FvGly. (A) Protein–protein interaction (PPI) network constructed with the STRING server (line thickness expresses the strength of data support). Binding modes of FvGly were obtained by molecular docking with (B) iNOS, (C) Akt1, and (D) Mapk14. Residues involved in interactions are highlighted, and hydrogen bonds are colored in yellow dashed lines.

### FvGly Reduces MAYV-Induced Disease in an Experimental *In Vivo* Mice Model

Considering the potential antiviral activity of FvGly, we sought to investigate the effect of FvGly treatment on MAYV-induced disease in a previously established mouse model.^22^ To this end, we treated 11- to 12-day-old mice daily with one dose of 20 mg/kg of FvGly or vehicle via intraperitoneal injection, starting 1 day before MAYV infection and following until 4 dpi, as indicated (Figure 6A). Infected mice presented a higher weight gain in the FvGly-treated group compared to untreated mice (Figure 6B). However, despite clinical improvement, the treatment did not result in a significant reduction in the footpad or muscle viral load, both of which are important sites of MAYV replication (Figure 6C,D). In agreement with the reduction of MAYV-induced disease, histological analysis of the mice’s right gastrocnemius muscle showed that the treatment preserved muscle fiber structure and strikingly reduced inflammation and lesion areas when compared with untreated mouse groups (Figure 6E). This indicates that, at 6 dpi, even without effectively controlling viral load through the treatment regimen and dose used, FvGly was able to protect against damage induced by the infection.

**Figure 6:**
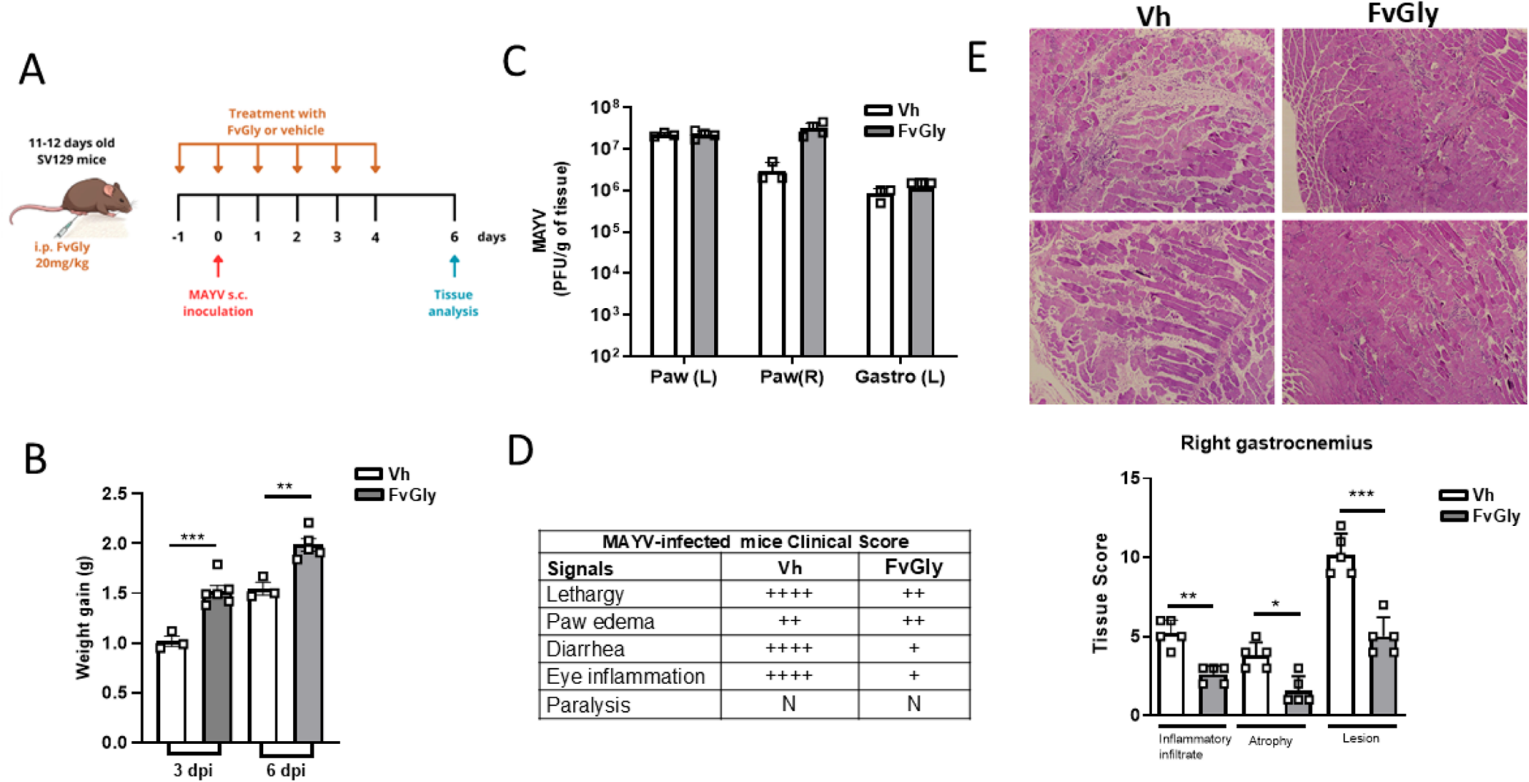
FvGly treatment reduced MAYV-induced disease in vivo. (A) Schematic representation of the experimental design. 11-day-old mice were treated intraperitoneally with one dose of 20 mg/kg of FvGly or vehicle (Vh), starting 1 day before (Day −1) MAYV inoculation in the left foot pad (Day 0) and daily (Day 0–5). Tissue samples were collected at 6 days post-infection (dpi). (B) Individual mice weight was monitored over the days, and weight gain was plotted in relation to the beginning of the experiment (Day −1). (C) Tissue viral load was determined by plaque assay from samples collected at 6 dpi. (D) Representation of observed clinical signal scores in MAYV-infected mice. Symbols represent the presence of signals in (+) Mild, (++) Moderate, and (++++) Severe form. *N* = not observed. (E) Right gastrocnemius muscle samples of Vh or FvGly groups were embedded in paraffin after dehydration, and 5-μm tissue sections were prepared, stained with H&E, and scored. Results are means ± SD of data from at least three independent experiments. Statistical analysis was performed using one-way ANOVA followed by Tukey’s multiple comparison test. *p < 0.05, **p < 0.01, ***p < 0.001 relative to Vh. The mice infection scheme (A) was created using free domain images.

## Discussion

Arboviruses circulate endemically around the globe, particularly in the region of the Americas, infecting thousands of people every year, increasing the demand for health care, overloading health systems, and causing social and economic impact.^23^ Moreover, the co-circulation of two or more arboviruses, which has been reported in many regions, can lead to coinfections, adding extra complexity to this scenario.^24,25,26^ Unfortunately, we still face a lack of approved and effective antiviral treatments for these viruses.

Several subclasses of flavonoids have been described as effective against arboviruses, including DENV, CHIKV, and ZIKV, such as the flavonol Quercetin, the flavone Baicalein/Baicalin, and the flavanone Pinocembrin.^27^ Flavanones with antiviral activity could be isolated from citrus fruits, but also from a variety of plants.^27^ Here, we used an FvGly isolated from *Faramea truncata*, but it could be easily obtained from different *Faramea* species, indicating its abundance in nature.^18,19^ Together with our previous studies,^21,22,28^ we expand FvGly’s antiviral activity to a robust anti-DENV, anti-ZIKV, and anti-MAYV effect in target cells. Similar to what was observed in our study, Pinocembrin, a 5,7-dihydroxy flavanone, was effective against DENV-2, ZIKV, and CHIKV by presenting a nonvirucidal post-entry activity,^29^ reinforcing the broad-spectrum potential use of the flavanone subclass.

The antiviral mechanism of flavonoids may involve a combination of direct effects on viral replication and modulation of host cell signaling involved in metabolism, antiviral response, and repair cascades.^11,12,27^ Both pre- and post-treatment strategies inhibited ZIKV and MAYV infectious particle release without a direct effect on particle infectivity, suggesting some indirect mechanism of FvGly. However, post-entry treatment seems to result in more efficient antiviral activity and in protecting cells from virus-induced cell death, and no additive effect was observed when we combined pre- and post-treatment strategies. Therefore, we could not exclude the possibility that FvGly also exerts an effect on viral proteins together with a modulation of cellular targets. Despite pre- and post-treatment not significantly reducing intracellular viral RNA levels, it impacts the amount of viral RNA released from MAYV- and ZIKV-infected cells and the E1MAYV-positive staining in cells, confirming FvGly antiviral activity by reducing both genomic RNA and viral protein synthesis.

The CHIKV and MAYV belong to the same *Alphavirus* genus, from the *Togaviridae* family.^2^ Curiously, FvGly was not efficient in inhibiting CHIKV infection in C2C12 cells. For CHIKV infection, the highest inhibitory effect, of about 45%, was observed when combining pre- and post-treatment. This may indicate that the replication efficiency of CHIKV overrides the modulatory effects of FvGly or that CHIKV is less dependent on the pathway modulated by FvGly than MAYV in myoblast C2C12 cells. Few groups have tested, in the same study and under the same experimental conditions, the activity of flavonoid subtypes against CHIKV and MAYV to allow efficiency comparison, but there is independent evidence of antiviral activity of some flavonoids against both viruses.^12^ For example, silymarin reduced the cytopathic effect and replication of MAYV and CHIKV, but studies were conducted treating HepG2 and Vero cell cultures, respectively, in addition to the use of different replication systems.^30,31^ Further studies exploring the FvGly mechanism of action, including other cell targets of alphavirus infection, would be important to understand the side effects and to propose improvements in its antiviral effect.

The *in silico* analyses performed suggested a prominent interaction of FvGly with the iNOS enzyme, as well as with the Akt and MAPK pathways. These enzymes are involved in closely related cascades that play a crucial role in several cellular signaling processes that regulate inflammation, response to stress, cell growth, and survival.^32,33,34^ It was previously demonstrated that the flavanone naringenin reduces nitrite production in lipopolysaccharide (LPS)-stimulated macrophages, evidencing that this flavanone could inhibit iNOS activity.^35^ Recently, we demonstrated that inflammation and reactive oxygen species induce persistent atrophy and skeletal muscle lesions in MAYV and CHIKV infections in young mice.^28^ Furthermore, treatment with Monomethyl Fumarate, an inducer of antioxidant genes via the Nrf2 pathway, was able to reduce inflammation, injury, and muscle atrophy induced by infection.^28^ However, the involvement of nitrogen species in oxidative stress induced by infection was not investigated. In agreement with anti-inflammatory and tissue-protective effects of FvGly, treatment of MAYV-infected young mice resulted in improved weight gain, clinical scores, and mainly reduced muscle lesions. Despite our data indicating that FvGly treatment reduces skeletal muscle inflammation induced by MAYV infection, this was not explored in the present manuscript. Further investigation is necessary to describe the mechanisms of action of FvGly and to confirm the anti-inflammatory properties of FvGly.

Protective effect of FvGly was observed even in the absence of the reduction in viral load in articular and muscular tissue, which are the main targets of alphavirus replication in this model.^22,28^ The low efficiency in controlling viral replication *in vivo* may be related to the delivery and availability of FvGly. Despite numerous findings reporting *in vitro* efficacy of flavonoids, poor bioavailability has been proposed to explain the low success of flavonoid compounds *in vivo* studies and new strategies have been proposed to overcome this limitation.^27^ In the present study, we administered one daily dose, but the bioavailability of FvGly was not evaluated. This parameter may explain the low efficiency to control viral load and could also be relevant for the development of alternative treatment strategies in mice.

Taken together, our results provide evidence of the broad-spectrum effectiveness of FvGly activity to control viral replication *in vitro* and to reduce damage induced by viral infection *in vivo*. This study contributes additional acknowledgment regarding the therapeutic properties of the flavonoid class and could be useful for further development and proposition of possible therapeutic strategies in the current alarming scenario for these arboviruses, especially in the Americas.

## Methods

### Virus Stocks

The MAYV (ATCC VR-66, strain TR 4675), CHIKV (BHI3745/H804709, kindly provided by Dr. Amilcar Tanuri), and ZIKV strain (BRPE243/2015, KX197192) were propagated in BHK-21 (ATCC-CCL-10) or C6/36 cells using a multiplicity of infection (MOI) of 0.01, as previously described,^21,28^ to produce viral stocks. After infection, culture mediums were collected and centrifuged at 2000 × *g* for 10 min to remove cellular debris, aliquoted, and stored at −80 °C. The same procedure was performed using uninfected cells to allow the production of the “Mock” stock. The titer of the viral stock was determined by plaque assay in Vero cells cultured in high-glucose Dulbecco’s modified Eagle medium (DMEM; Invitrogen) after 10-fold serial dilutions.

### Plant Material and Isolation of the Bioactive Flavanone Glycoside

*Faramea truncata* was collected in May 2014 in the Parque Nacional da Serra dos Órgãos, Guapimirim, Rio de Janeiro, Brazil. A voucher specimen was deposited at the Herbarium of the Instituto de Biologia, Universidade Federal do Rio de Janeiro (RFA 40642). The collection was authorized by SISBIO-ICMBio-MMA Brazil (no. 46504-2). The species was identified by botanist Mário Gomes. A crude methanolic extract of *F. truncata* leaves was defatted and subjected to solid-phase extraction using C18 silica gel cartridges for the isolation of the compound FvGly, identified as 2S-5-hydroxy-4′-methoxy-flavanone-7-*O*-β-D-apiofuranosyl-(1→6)-β-d-glucopyranoside, according to a previously reported method.^18^ Following these procedures, isolation was confirmed by high-performance liquid chromatography coupled to a diode-array detector (HPLC-DAD), and the purity of FvGly was estimated to exceed 95%. Structural characterization was performed by nuclear magnetic resonance (NMR) spectroscopy. Stock solutions of FvGly were prepared in dimethyl sulfoxide (DMSO).

### Cell Culture, Infections, and Treatment

SH-SY5Y cells (human neuroblastoma cell lineage, kindly provided by Dr. Luciana Costa from Instituto de Microbiologia of UFRJ), were cultured in DMEM/F12 medium supplemented with 10% FBS, and C2C12 cells (murine myoblasts cell lineage; ATCC, CRL-1772), kindly donated by Dr. Flávia Bloise (IBCCF-UFRJ, Brasil), were cultured in DMEM High Glucose medium. Both cell lines were maintained at 37 °C with 5% CO_2_. For the assays, cells were plated in 48-well plates and infected with ZIKV for SH-SY5Y cells, and MAYV or CHIKV for C2C12 cells, with an MOI of 0.1 for 1 h (adsorption time) and then cultured for respective times until analysis.

Treated conditions were performed using FvGly diluted in the respective culture medium with 5% FBS at the desired concentrations. The highest final concentration of DMSO was used as a vehicle (Vh) condition in the assays. For cytotoxicity analysis, cells were treated with increasing concentrations of FvGly for 24 h; in pre-infection treatment, cells were treated 24 h before infection, and then viral infection was performed using fresh medium with 5% FBS without FvGly. For post-infection treatment, after the virus adsorption time, the medium was replaced with one containing FvGly with 5% FBS. Mock-infected cells were subjected to the same procedures and treated with Vh or left untreated (not treated; NT). FvGly cytotoxicity and cell viability after infection were determined using MTT [3-(4,5-dimethylthiazol-2-yl)-2,5-diphenyltetrazolium bromide; Life Technologies] metabolization. The culture medium was collected to quantify the infectious particles released into the medium by a plaque assay, and % inhibition was determined using the viral titer of the Vh condition as reference.

### Viral RNA Quantification

Intracellular and released viral genomes were quantified by real-time quantitative PCR. Total RNA was extracted from the culture supernatant or from infected cells using the TRIzol Reagent (Invitrogen) according to the manufacturer’s protocol. The purity and integrity of RNA were determined by 260/280 and 260/230 nm absorbance ratios. Isolated RNA was subjected to DNase treatment (Ambion DNase I, Thermo Fisher, Waltham, MA, USA) and then reverse-transcribed using a High-Capacity cDNA Reverse Transcription Kit (Applied Biosystems, Waltham, MA, USA). Detection of genomic RNA of ZIKV and MAYV by real-time PCR analysis was conducted using the TaqMan Mix kit with specific dyes (#4304437, Applied Biosystems, Foster City, CA, USA), as previously described.^21,28^ For the MAYV analysis, we used MAYV forward (5′-CCT TCA CAC AGA TCA GAC-3′), MAYV reverse (5′-GCC TGG AAG TAC AAA GAA-3′), and the probe MAYV–FAM (5′-/56-FAM/CAT AGA CAT CCT GAT AGA CTG CCA CC/3BHQ_1/-3′). For ZIKV analysis, we used ZIKV_1162C forward (5′-CCG CTG CCC AAC ACA AG-3′), ZIKV_1086 reverse (5′-CCA CTA ACG TTC TTT TGC AGA CAT-3′), and the probe ZIKV_1107_fam (5′-/56-FAM/AGC CTA CCT TGA CAA GCA GTC AGA CAC TCA A/3BHQ_1/-3′). Cycle threshold values were used to calculate the equivalent (eq) of PFU/μg of total RNA after conversion using a standard curve with serial 10-fold dilutions of ZIKV or MAYV.

### Immunofluorescence Assay

C2C12 cells were cultured in a 24-well plate until reaching confluency and then infected with MAYV (MOI 0.1). After 24 h of infection, supernatants were removed, and cells were fixed with 4% paraformaldehyde. Cells were then washed with PBS, permeabilized with 0.1% saponin (Vetec) in 0.1% BSA (Sigma) in PBS for 20 min, and blocked with 1% BSA in PBS with 2% FBS for 1 h. Primary antibodies for alphavirus E proteins (monoclonal mouse anti-Eastern equine encephalitis, Millipore) were used at a dilution of 1:1000, followed by incubation with 1:500 secondary antibody Alexa Fluor 488 anti-mouse (Invitrogen) for 30 min. Infected cells were visualized in a fluorescence microscope (IX81– Olympus) with 20× magnification. Positive cells were determined by counting DAPI-positive nuclei marked with viral antigens, and fluorescence in the field was quantified using ImageJ software (version 1.51j8).

### *In Silico* Target Fishing and Molecular Docking

For the mechanistic investigation through target fishing, the 2D structure of FvGly was built using ACD/ChemSketch (Advanced Chemistry Development, Inc., Toronto, ON, Canada) and exported as Simplified Molecular Input Line Entry Specification (SMILES) format to be used as input data in the servers TargetHunter (https://www.cbligand.org/TargetHunter), Similarity Ensemble Approach (SEA) (http://sea.bkslab.org/), Binding Database (BDB) (https://www.bindingdb.org/bind/index.jsp), and CHEMBL (https://www.ebi.ac.uk/chembldb). For PharmMapper (http://www.lilab-ecust.cn/pharmmapper/), the structure was exported in mol2 format to enable human target screening for human target search. The overlapping potential targets that showed ligand structural similarity higher than 90% were extracted from the servers and submitted to STRING version 11.5 (https://string-db.org) for the protein–protein interaction analysis by their gene names in the *Homo sapiens* organism, with a minimum required interaction score of 0.4 for network generation.

The molecular docking study with potential targets was performed after validation by the redocking approach. The three-dimensional (3D) structure of the flavonoid at pH 7.4 was built and optimized using Avogadro software.^36^ The 3D structures of iNOS, Akt, and MAPK were obtained from the Protein Data Bank (PDB) under codes 1NSI, 1UNQ, and 1W82, respectively.^37,38,39^ The cocrystallized ligand and solvent molecules were removed with PyMOL 1.3 (The PyMOL Molecular Graphics System, Version 1.3, Schrödinger, LLC, San Francisco, CA, USA). The molecular docking studies were performed in the AutoDock 4.2 program running on a Windows-based PC, and the docking files were prepared using AutoDock Tools.^40^ The macromolecules were treated as rigid; polar hydrogen atoms were added; nonpolar hydrogen atoms were merged; and Gasteiger charges were assigned by default. The grid center was established on the ligand site, with 60 × 60 × 60 points for iNOS and MAPK and 50 × 50 × 50 for Akt, with 0.375 Å spacing. Docking studies were conducted using the semiempirical free energy function and the Lamarckian Genetic Algorithm (LGA). LGA default parameters such as initial population (150), number of energy assessments (25,00,000), mutation rate (0.02), crossover rate (0.8), and elitism (1) were maintained while a total of 50 independent docking runs were carried out using the program’s default parameters. The results of the most favorable free energies of binding in the most populated clusters were selected as possible structures of the resultant complexes.

### Analysis of the Effect of FvGly in a MAYV-Induced Disease Mice Model

The experiments were performed using WT SV129 mice 12 days after birth. Treatment with FvGly or vehicle started 1 day before the infection and continued daily until 4 days after the infection. Mice were treated via intraperitoneal injection with a dose of 20 mg of FvGly/kg of animal, in a final volume of 50 μL. The untreated group received only 50 μL of vehicle (relative amount of DMSO). Mice were subcutaneously inoculated in the left footpad with 10^5^ PFU of MAYV, using a final volume of 20 μL. Young mice were housed with the uninfected mother during the entire experiment in polypropylene cages with free access to chow and water. Both groups were weighed daily, and clinical signs were recorded. The area of hind limb foot edema in the animals was determined from the width-height measurements of the metatarsal region using a digital caliper. Tissue samples were collected at 6 days post-infection and stored at −80 °C until viral load determination by plaque assay or fixed in 4% formaldehyde. Fixed right gastrocnemius muscles were embedded in paraffin after dehydration. Paraffin-embedded tissue sections of 5 μm were prepared and stained with hematoxylin and eosin (H&E). Images were obtained using optical microscopy with a magnification of 10X (Olympus BX40), and images were acquired using Leica Application Suite 3.8 software. For scoring, H&E muscle tissue images of MAYV-infected mice were numerically classified according to the intensity of cellular infiltrate, fiber atrophy, and lesion area by a researcher blind to the experimental condition. All experimental procedures were performed following protocols and standards established by the National Council for Control of Animal Experimentation (CONCEA, Brazil) and approved by the Institutional Animal Care and Use Committee (CEUA) at the Federal University of Rio de Janeiro (protocol no. A04/22-036-18; CEUA-UFRJ, Rio de Janeiro, Brazil).

### Statistical Analysis

Statistical analyses were performed using GraphPad Prism version 8.0.2 for Windows (GraphPad Software, La Jolla, CA). The tests used are indicated in the corresponding figure legends. Briefly, multiple groups were analyzed using one-way analysis of variance (ANOVA), followed by multiple-comparison analyses.

## Supplementary Material



## Notes

Additional experimental results of tests on the combined effect of pre- and post-treatment strategies (Figure S1), the virucidal activity of FvGly (Figure S2), and a table with the complete list of identified targets of FvGly by the target fishing method (Table S1) (PDF)
