# Supplementary material for: Bioactive Flavanone Glycoside Isolated from Leaves of Faramea Species Presents Antiviral and Protective Activity against Zika and Mayaro Virus Infection
Source: ACS Omega. 2025 Dec 3;10(49):60196–204. doi: 10.1021/acsomega.5c06047 (PMC12713426; doi:10.1021/acsomega.5c06047)
Supplement: Supplementary file 1 [file ao5c06047_si_001.pdf]

Bioactive flavanone glycoside isolated from leaves of *Faramea* species presents antiviral and protective activity against Zika and Mayaro virus infection

Iris Paula Guimarães-Andrade[1], Rodolfo S. Barboza[2], Mariana Oliveira Lopes da Silva[1], Raissa Alves da Conceição[3], Nathalia Carraio de Albuquerque[1], Daniel Gavino-Leopoldino[1], Romulo Leão da Silva Neris[1], Alessandra Mendonça Teles de Souza[3], Ligia M. M. Valente[2], Iranaia Assunção-Miranda[1]*

1. LaRIV, Department of Virology, Instituto de Microbiologia Paulo de Góes, Universidade Federal do Rio de Janeiro (UFRJ), Rio de Janeiro 21941-902, Brazil
2. Instituto de Química, Universidade Federal do Rio de Janeiro (UFRJ), Rio de Janeiro 21941-909, Brazil
3. Laboratory of Molecular Modeling & QSAR Faculty of Pharmacy, Universidade Federal do Rio de Janeiro, Rio de Janeiro 21.944-970, RJ, Brazil

*Correspondence: iranaiamiranda@micro.ufrj.br

Figure S1

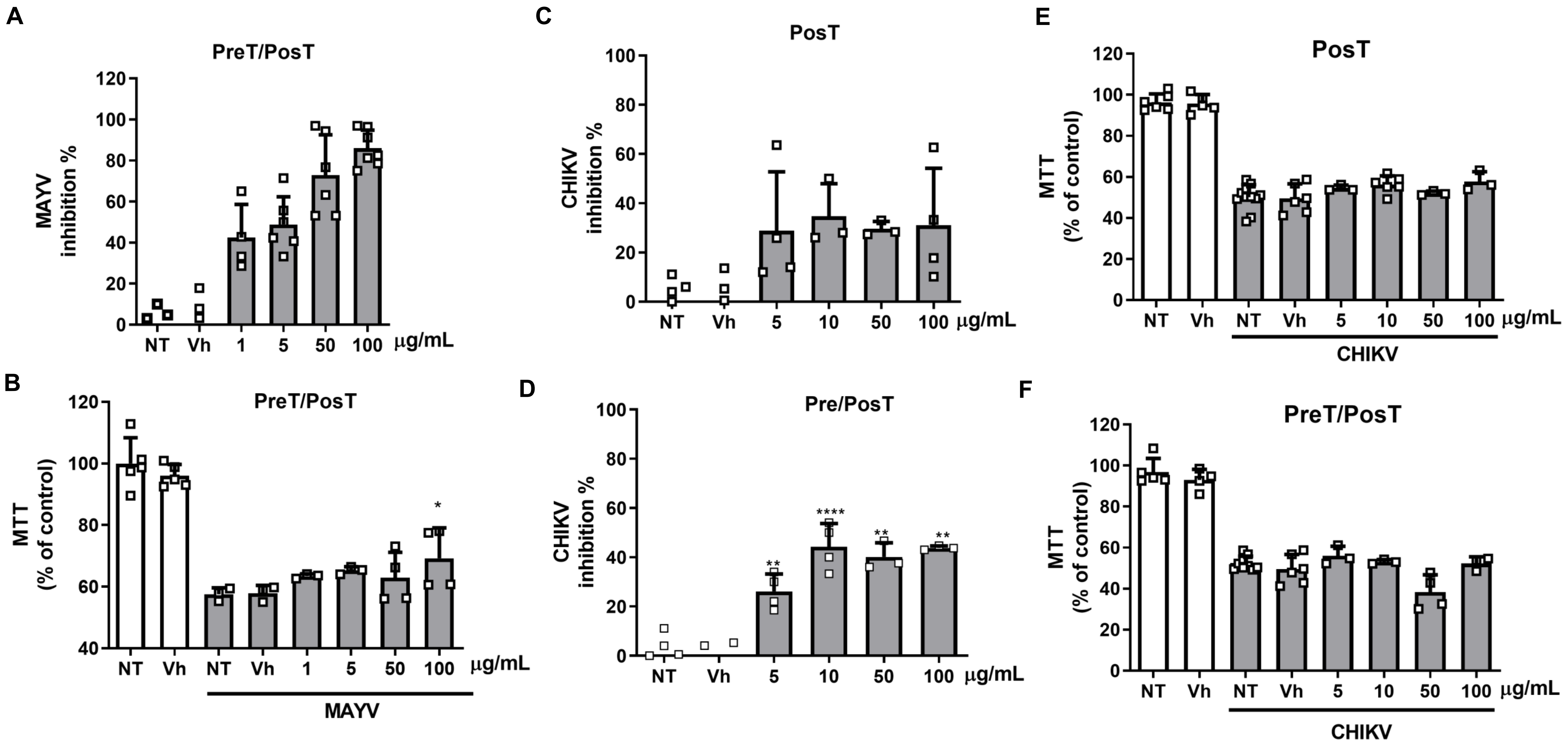

A
PreT/PosT
MAYV inhibition %
120
100
80
60
40
20
0
NT
Vh
1
5
50
100
μg/mL
B
PreT/PosT
MTT (% of control)
120
100
80
60
40
*
NT
Vh
NT
Vh
1
5
50
100
μg/mL
MAYV
C
PosT
CHIKV inhibition %
100
80
60
40
20
0
NT
Vh
5
10
50
100
μg/mL
D
Pre/PosT
CHIKV inhibition %
100
80
60
40
20
0
**
****
**
**
NT
Vh
5
10
50
100
μg/mL
E
PosT
MTT (% of control)
120
100
80
60
40
20
0
NT
Vh
NT
Vh
5
10
50
100
μg/mL
CHIKV
F
PreT/PosT
MTT (% of control)
120
100
80
60
40
20
0
NT
Vh
NT
Vh
5
10
50
100
μg/mL
CHIKV

Figure S1. Effect of FvGly on MAYV and CHIKV infection by combining Pre- and Pos-treatment strategies. Myoblast cell lineage (C2C12) culture was treated with increasing concentrations of FvGly after (Pos-T) or combining pre and post-treatement (PreT/PosT) on MAYV or CHIKV infection, as indicated at figure. (**A,C and D**) Quantification of infectious particles released was determined by plaque assay and effect of FvGly treatment was determined in % of inhibition using viral titer of vehicle (Vh) condition as reference. (**B, E and F**) C2C12 cell viability was determined by MTT assay after 48h hour of infection treated with Vh or FvGly in PosT or combining PreT/PosT strategies, as indicated at figures. Results are means ± SD of data from at least three independent experiments. Statistical analysis was performed using one-way Anova followed by Turkey´s multiple comparison test. * represents $p < 0.05$, ** = $p < 0.01$ and **** = $p < 0.0001$ relative to Vh.

Figure S2

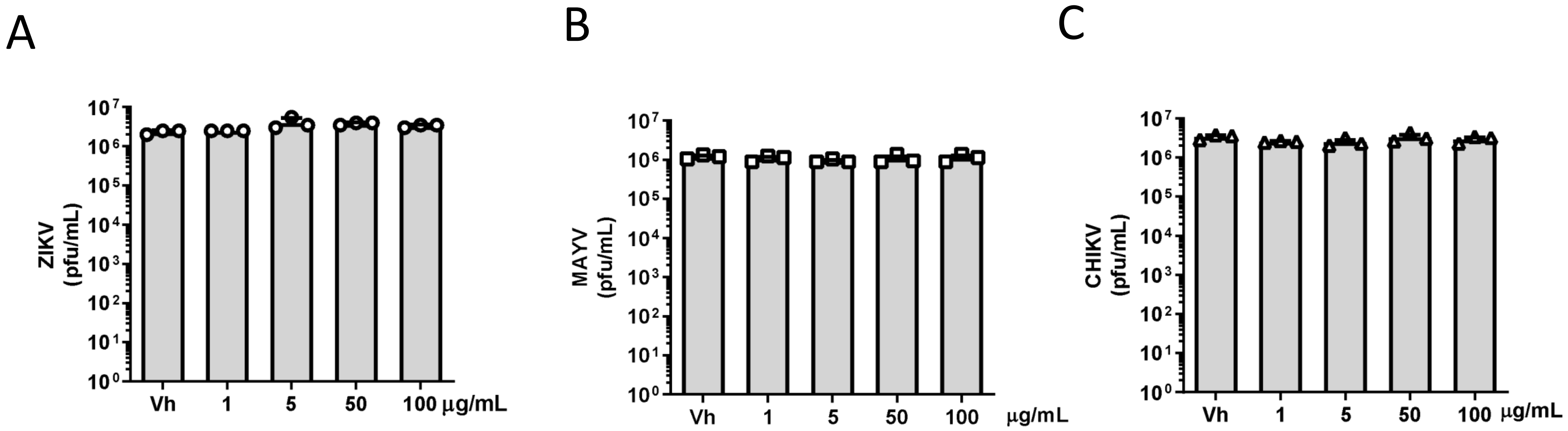

Figure S2. Test of Virucidal Activity of FvGly. Indicated concentrations of FvGly or highest dose of Vehicle (Vh) were prepared in medium and tested to virucidal activity by direct incubation with 10^5 PFU of ZIKV (A), MAYV (B) and CHIKV (C) at 37°C with 5% CO_2 for 1 hour. After this period, viral infectivity after treatment was determined by plaque assay in VERO cells.

Table S1. Potential targets of FvGly identified by target fishing method.

Protein	Gene	Classification	PDB
**iNOS**	NOS2	Oxidoredutase	1NSI
**Coagulation factor X**	F10	Hydrolase	3ENS
**Methionine aminopeptidase 2**	METAP2	Hydrolase	1QZY
**Cathepsin K**	CTSK	Hydrolase	1TU6
**Inosine-5'-monophosphate dehydrogenase2**	IMPDH2	Oxidoredutase	1NFB
**Dihydrofolate reductase**	DHFR	Oxidoreductase	1PD8
**Farnesyl pyrophosphate synthase**	FDPS	Transferase	1YQ7
**Mitogen-activated protein kinase 14**	MAPK14	Kinase	1W82
**Focal adhesion kinase1**	PTK2	Transferase	1MP8
**Adenosine kinase**	ADK	Transferase	1BX4
**Serine/threonine-protein kinase AKT1**	AKT1	Transferase	1UNQ
**Butyrylcholinesterase**	BCHE	Hydrolase	1XLW
**Carbonic anhydrase 2**	CA2	Lyase	1G53
**Aldo-keto reductase family 1 member C2**	AKR1C2	Reductase	1IHI
